# Clinical and genetic characteristics of 15 families with hereditary hypophosphatemia: Novel Mutations in *PHEX* and *SLC34A3*

**DOI:** 10.1371/journal.pone.0193388

**Published:** 2018-03-05

**Authors:** Sezer Acar, Huda A. BinEssa, Korcan Demir, Roua A. Al-Rijjal, Minjing Zou, Gönül Çatli, Ahmet Anık, Anwar F. Al-Enezi, Seçil Özışık, Manar S. A. Al-Faham, Ayhan Abacı, Bumin Dündar, Walaa E. Kattan, Maysoon Alsagob, Salih Kavukçu, Hamdi E. Tamimi, Brian F. Meyer, Ece Böber, Yufei Shi

**Affiliations:** 1 Division of Pediatric Endocrinology, Dokuz Eylul University School of Medicine, Izmir, Turkey; 2 Department of Genetics, King Faisal Specialist Hospital & Research Centre, Riyadh, Saudi Arabia; 3 Division of Pediatric Endocrinology, Katip Çelebi University School of Medicine, Izmir, Turkey; 4 Division of Pediatric Endocrinology, Adnan Menderes University School of Medicine, Izmir, Turkey; 5 Department of Endocrinology, Dokuz Eylul University School of Medicine, Izmir, Turkey; 6 Division of Pediatric Nephrology, Dokuz Eylul University School of Medicine, Izmir, Turkey; Sant Joan de Déu Children's Hospital, SPAIN

## Abstract

**Background:**

Hereditary hypophosphatemia is a group of rare renal phosphate wasting disorders. The diagnosis is based on clinical, radiological, and biochemical features, and may require genetic testing to be confirmed.

**Methodology:**

Clinical features and mutation spectrum were investigated in patients with hereditary hypophosphatemia. Genomic DNA of 23 patients from 15 unrelated families were screened sequentially by PCR-sequencing analysis for mutations in the following genes: *PHEX*, *FGF23*, *DMP1*, *ENPP1*, *CLCN5*, *SLC34A3* and *SLC34A1*. CytoScan HD Array was used to identify large deletions.

**Results:**

Genetic evaluation resulted in the identification of an additional asymptomatic but intermittent hypophosphatemic subject. Mutations were detected in 21 patients and an asymptomatic sibling from 13 families (86.6%, 13/15). *PHEX* mutations were identified in 20 patients from 12 families. Six of them were novel mutations present in 9 patients: c.983_987dupCTACC, c.1586+2T>G, c.1206delA, c.436+1G>T, c.1217G>T, and g.22,215,887–22,395,767del (179880 bp deletion including exon 16–22 and *ZNF645*). Six previously reported mutations were found in 11 patients. Among 12 different *PHEX* mutations, 6 were de novo mutations. Patients with de novo *PHEX* mutations often had delayed diagnosis and significantly shorter in height than those who had inherited *PHEX* mutations. Novel compound heterozygous mutations in *SLC34A3* were found in one patient and his asymptomatic sister: c.1335+2T>A and c.1639_1652del14. No mutation was detected in two families.

**Conclusions:**

This is the largest familial study on Turkish patients with hereditary hypophosphatemia. *PHEX* mutations, including various novel and de novo variants, are the most common genetic defect. More attention should be paid to hypophosphatemia by clinicians since some cases remain undiagnosed both during childhood and adulthood.

## Introduction

Hereditary hypophosphatemia or hypophosphatemic rickets (HR) when it occurs in children is a group of rare renal phosphate-wasting disorders with a prevalence of 3.9 per 100,000 live births [1]. It is characterized by hypophosphatemia and bone mineralization defects such as rickets and osteomalacia [2–4]. The patients usually present with a spectrum of clinical features such as growth retardation with disproportioned short stature, enlargement of growth plates of rapidly growing bones, lower limb deformities, bone pain, loss of teeth, and dental abscess.

Various genetic defects are known to cause the disease [5–7]. Among them, inactivating mutations in the *PHEX* (phosphate regulating gene with homologies to endopeptidases on the X chromosome, MIM 300550) lead to X-linked HR (XLHR, MIM 307800), the most common form of hereditary hypophosphatemia [8]. More than 424 different mutations have been listed in the Human Gene Mutation Database (HGMD, http://www.hgmd.cf.ac.uk/ac/index.php, accessed on Jan 17, 2018). Gain-of-function mutations at codon R176 or R179 in the proteolytic cleavage domain of *FGF23* (fibroblast growth factor 23, MIM 605380) cause autosomal dominant HR (ADHR, MIM 193100) [9, 10]. Five different mutations are currently listed in the HGMD (accessed on Jan 17, 2018). Inactivating mutations in the *DMP1* (dentin matrix acidic phosphoprotein 1, MIM 600980) or *ENPP1* (ectonucleotide pyrophosphatase/phosphodiesterase 1, MIM 173335) result in autosomal recessive HR type 1 (ARHR1, MIM 241520) [11, 12] or autosomal recessive HR type 2 (ARHR2, MIM 613312) [13], respectively. The frequency of ARHR1 and ARHR2 is about the same. Currently, there are 9 different *DMP1* and 8 *ENPP1* mutations listed in the HGMD (accessed on Jan 17, 2018). Interestingly, *ENPP1* mutations are more frequently reported in patients with idiopathic infantile arterial calcification or generalized arterial calcification of infancy (49 different mutations are listed in the HGMD), suggesting that a different pathway is involved in the generation of ARHR2 [14]. Inactivating mutations in the *SLC34A1* (sodium-dependent phosphate cotransporter 2a, MIM 182309) give rise to either autosomal recessive hypophosphatemic nephrolithiasis/osteoporosis-1 (MIM 612286) [15, 16] or Fanconi renotubular syndrome-2 (MIM 613388) [17], whereas a loss-of-function mutation in the *SLC34A3* (sodium-dependent phosphate cotransporter 2c, MIM 609826) results in hereditary HR with hypercalciuria (HHRH, MIM 241530) [18, 19]. *SLC34A1* mutation also causes autosomal-recessive infantile hypercalcemia-2 (HCINF2, MIM 616963) due to renal phosphate wasting-induced overproduction of 1,25(OH)_2_D_3_ [20]. Unlike inactivation mutation in *CYP24A1* (MIM 126065) which causes infantile hypercalcemia-1 (HCINF1) [21], patients with HCINF2 respond rapidly to phosphate supplementation [20]. Currently, there are 25 different *SLC34A1* and 33 *SLC34A3* mutations listed in the HGMD (accessed on Jan 17, 2018). In addition, inactivating mutations in the *CLCN5* (chloride voltage-gated channel 5, MIM 300008) lead to X-linked recessive HR (XLRHR, MIM 300554) and may be part of clinical presentations in Dent disease 1 (MIM 300009) characterized by low molecular weight proteinuria, hypercalciuria, nephrocalcinosis, nephrolithiasis, and renal failure [22–24]. More than 250 different *CLCN5* mutations are listed in the HGMD (accessed on Jan 17, 2018).

PHEX and DMP1 negatively regulate the expression of FGF23, a novel phosphaturic hormone produced in osteocytes, which is secreted into the circulation after undergoing O-glycosylation by GALNT3 [25, 26]. FGF23 is a key regulator of phosphate reabsorption and 1,25(OH)_2_D_3_ synthesis in the proximal renal tubules [27, 28]. FGF23 levels are increased in patients with XLHR, ADHR, ARHR1, and ARHR2 leading to phosphaturia, low serum phosphate, and low serum 1,25(OH)_2_D_3_ level due to its inhibition of vitamin D 1-α hydroxylase. However, in patients with HCINF2, HHRH, and XLRHR, FGF23 levels are normal or low-normal, suggesting an FGF23-independent renal tubular defect [29]. In contrast to patients with XLHR, ADHR or ARHP, who are usually treated with high doses of alphacalcidol or calcitriol and multiple daily doses of oral phosphate, oral phosphate supplementation alone is sufficient for patients with HHRH.

In our previous studies, 27 patients from 16 unrelated Turkish families were investigated [30–32]. In the present study, we analyzed clinical and genetic characteristics of additional patients from 15 unrelated families from a different region of Turkey.

## Subjects and methods

### Patients

Parents and available siblings of index patients (n = 51) from 15 unrelated Turkish families were enrolled to the study. Hereditary hypophosphatemia was diagnosed based on clinical and laboratory assessment. The clinical and laboratory data at the time of diagnosis were summarized in Table 1. The cases suspected of having renal failure, malabsorption, renal tubular acidosis and other tubulopathies that cause secondary hypophosphatemia (e.g., Fanconi’s syndrome, glycogen storage disease, and galactosemia), vitamin D-dependent rickets type 1 and 2 were excluded from the study. This study was approved by the institutional review board of the Dokuz Eylul University Faculty of Medicine. Written informed consent was obtained from the children and their parents.

**Table 1:** Summary of clinical and laboratory findings in patients with hereditary hypophosphatemia.

Family	Subjects	Clinical features	Age (years)	Height SDS	Ca mg/dL	P mg/dL	ALP IU/L	25OHD3 ng/ml	1,25(OH)_2_ D3 pg/mL	PTH ng/L	TPR	TmP/GFR
I	I-1 Father	normal	38	-1.37	ND	ND	ND	ND	ND	ND	ND	ND
	I-2 Mother	normal	38	0.56	ND	ND	ND	ND	ND	ND	ND	ND
	I-3 Daughter	Inability to walk, genu varum deformity	2.4	-3.76	9.2	2.5	829	21.2	50.8	94	56.9	1.42
II	II-1 Father	normal	38	-0.62	ND	ND	ND	ND	ND	ND	ND	ND
	II-2 Mother	Short stature, genu varum deformity	37	-3.95	9.8	2.1	111	10.5	ND	73	87	1.70
	II-3 Daughter	Short stature,genu varum deformity	2	-2.09	10.2	2.1	531	31.1	ND	52.5	62.3	1.31
III	III-1 Father	normal	42	-0.26	ND	ND	ND	ND	ND	ND	ND	ND
	III-2 Mother	Genu varum,short stature	40	-3.28	10.1	1.7	106	ND	ND	ND	ND	ND
	III.3 Daughter	Inability to walk,genu varum deformity	5	-2.13	8.5	2.2	667	20	30	319	67.8	1.49
	III-4 Son	Inability to walk, genu varum deformity	3.2	-1.58	9.2	3.1	600	80	ND	107	69.1	2.1
IV	IV-1 Father	normal	49	-1.65	ND	ND	ND	ND	ND	ND	ND	ND
	IV-2 Mother	normal	40	0.26	ND	ND	ND	ND	ND	ND	ND	ND
	IV-3 Daughter	Short stature, genu varum deformity	5	-3.33	9.4	2.0	559	28.3	ND	138	79.6	1.59
V	V-1 Father	Fractures, genu varum deformity	30	-3.71	9.6	2.1	143	23.7	ND	40	69.9	1.46
	V-2 Mother	normal	32	-1.28	ND	ND	ND	ND	ND	ND	ND	ND
	V-3 Daughter	Short stature, widening of wrist	1.3	-2.80	10	3.4	465	>70	ND	48.1	60.6	2.06
VI	VI-1 Father	normal	34	ND	ND	ND	ND	ND	ND	ND	ND	ND
	VI-2 Mother	normal	37	2.05	ND	ND	ND	ND	ND	ND	ND	ND
	VI3 Daughter	normal	8	ND	ND	ND	ND	ND	ND	ND	ND	ND
	VI-4 Son	Inability to walk,genu varum deformity	3.5	-3.76	9.8	2.8	925	35	ND	62.2	69.7	1.95
VII	VII-1 Father	normal	35	0.03	ND	ND	ND	ND	ND	ND	ND	ND
	VII-2 Mother	normal	27	0.26	ND	ND	ND	ND	ND	ND	ND	ND
	VII-3 Son	Inability to walk,genu varum deformity	4	-3.44	9.0	3.1	393	4.9	ND	154.7	58.7	1.82
VIII	VIII-1 Father	normal	35	-0.51	ND	ND	ND	ND	ND	ND	ND	ND
	VIII-2 Mother	normal	33	-0.26	ND	ND	ND	ND	ND	ND	ND	ND
	VIII-3 Daughter	Short stature	4.5	-3.89	9.8	2.4	581	39.1	130	71.5	78.5	1.88
IX	IX-1 Father	normal	36	-0.95	ND	ND	ND	ND	ND	ND	ND	ND
	IX-2 Mother	Short stature, genu varum	36	-2.35	9.65	1.83	81	15.7	ND	130	95.5	1.69
	IX-3 Son	Genu varum deformity	2.1	-0.75	10.1	2.6	827	32.3	ND	107.1	74	1.92
X	X-1 Father	normal	39	-0.54	ND	ND	ND	ND	ND	ND	ND	ND
	X-2 Mother	normal	33	-2.05	ND	ND	ND	ND	ND	ND	ND	ND
	X-3 Son	Nephrolithiasis	5	-0.54	9.8	2.5	548	12.5	27.5	33.9	85	2.1
	X-4 Daughter	Normal	6.9	-0.23	10.3	3.6	325	27.5	ND	13	82.5	2.97
XI	XI-1 Father	Normal	31	-0.25	ND	ND	ND	ND	ND	ND	ND	ND
	XI-2 Mother	Normal	28	-0.56	ND	ND	ND	ND	ND	ND	ND	ND
	XI-3 Son	Genu varum deformity	2.5	-0.4	10.3	3.2	434	6.8	ND	16.9	70	1.1
	XI-3 Son	Normal	6.2	ND	ND	ND	ND	ND	ND	ND	ND	ND
XII*	XII-1 Father	normal	41	0.72	9.59	3.73	78	ND	ND	30.1	ND	ND
	XII-2 Mother	normal	44	-1.46	9.52	2.12	114	ND	ND	61.2	ND	ND
	XII-3 Son	Genu varum deformity	2.9	-0.90	9.6	2.9	623	52	ND	62	74.9	2.29
	XII-4 Daughter	Genu varum deformity	2.9	1.18	9.8	2.9	401	35.1	ND	71.4	77	2.48
XIII	XIII-1 Father	Normal	56		9.89	2.76	61	22.2	ND	50.8	88	2.43
	XIII-2 Mother	Normal	53		9.73	3.23	98	10.9	ND	72	93.4	3.01
	XIII-3 Son	Inability to walk, rickets	7.5	-0.75	9.9	2.1	2533	ND	36.3	58	82	1.74
XIV	XIV-1 Father	Normal	36	-0.2	8.7	3.7	77	39.9	ND	63	93	3.4
	XIV-2 Mother	Shortstature, genu varum deformities	35	-2.8	8.9	2.3	44	< 8	ND	95	97	2.2
	XIV-3 Daughter	Short stature, genu varum deformities	2.7	-2.01	9.4	2.2	364	27	ND	49	75	1.65
XV	XV-1 Father	Normal	25	-1.13	ND	ND	ND	ND	ND	ND	ND	ND
	XV-2 Mother	Normal	28	-0.40	ND	ND	ND	ND	ND	ND	ND	ND
	XV-3 Daughter	Short stature, genu varum deformities	3.3	-3.04	9.4	2.9	677	107	54.2	79.3	83.4	2.8
	XV-4 Sister	Normal	6.9	-0.02	10.2	4.96	176	21.5	ND	11.4	92.3	4.5
Normal range					8.5–10.5	3.7–5.6^1^	90–325^2^	20–100	17–53	15–65	>85%	2.9–6.1^3^

*from a consanguineous family

ND: not done; SDS: standard deviation score or Z-score

SI unit conversions: to convert the values for 25OHD to nmol/L, multiply by 2.5; to convert the values for 1,25(OH)_2_D to pmol/L, multiply by 2.4

to convert the value for calcium to mmol/L, divide by 4; to convert the values for phosphate to mmol/L, divide by 3.1.

^1^ Normal range of serum phosphate is 4.8–8.2 mg/dL for 0–5 days of age, 3.8–6.5 mg/dL for 1–3 years of age, 3.7–5.6 mg/dL for 4–11 years of age, 2.9–5.4 mg/dL for 12–18 years of age and 2.7–4.5 mg/dL for adults.

^2^ Normal range of serum ALP is 80–380 IU/L for 0–1 year, 90–325 IU/L for 1–9 years, 82–380 IU/L for 9–18 years, 41–120 IU/L for over 18 years.

^3^ The normal ranges of TmP/GFR (mg/dL) vary with age: Birth, 3.6–8.6; 3 months of age, 3.7–8.25; 6 months of age, 2.9–6.5; 2–15 years of age, 2.9–6.1; adult, 2.2 to 3.6 mg/dL.

### Genomic DNA isolation

Genomic DNA from peripheral blood leukocytes was isolated using Gentra Blood Kit (Qiagen Corp, CA).

### DNA amplification and sequencing

DNA samples were first analyzed for mutations in all the coding exons and intron-exon boundaries of *PHEX*. If no mutations were identified, we then screened for mutations in the *FGF23* followed by consecutive analysis of *DMP1*, *ENPP1*, *CLCN5*, *SLC34A3* and *SLC34A1*. PCR primers and conditions were described previously[11, 13, 22, 30, 33, 34]. The resulting PCR products were directly sequenced using an automated ABI PRISM 3700 sequencer (Foster City, CA) or cloned into TA vector (Invitrogen, CA). Individual clones were subsequently sequenced.

### Identification of a large *PHEX* deletion

Copy number variation in genomic DNA was analyzed by CytoScan HD Array according to the manufacturer’s procedure (Affymetrix, Santa Clara, CA). The closest undeleted probes to the boundary of deleted region (copy number loss) in the *PHEX* gene were used as a starting point to map the breaking point. A total of 6 walking primers were used to perform PCR until a specific DNA fragment was amplified. The PCR products were directly sequenced.

### RNA isolation and RT-PCR

Total RNA from peripheral blood leukocytes was extracted as described previously [35]. Two μg of total RNA were reverse-transcribed into cDNA using Promega reverse transcription system (Promega, Madison, WI). To determine the effect of a splice site mutation at c.1586+2T>G, RT-PCR was used to amplify *PHEX* transcripts using the following two primers: 5’- CTATCCAGAGTTTATAATGAA-3’ (forward primer located in exon 13), and 5’- CTGTTCCCCAAAAGAAAGGCTT-3’ (reverse primer located in exon 16). The PCR conditions were 95°C for 5 minutes followed by 35 cycles of amplification (95°C for 1 min, 54°C for min, and 72°C for 1 min). The resulting PCR products were analyzed by gel electrophoresis and subsequently sequenced.

### *In silico* analysis of variants

The significance of single nucleotide variant (SNV) was predicted using the following four web-based programs: Mutation Taster (http://www.mutationtaster.org), PolyPhen-2 (http://genetics.bwh.harvard.edu/pph2/), SIFT (http://sift.jcvi.org/), and PROVEAN (http://provean.jcvi.org/index.php). SNV was considered to be mutation if they had: low allelic frequency (<0.01) in the normal population database; predicted to be damaging or disease-causing by at least three of four prediction programs; and present only in the patient or in strict segregation with phenotype in familial cases. Biallelic or compound heterozygous mutations were considered as disease-causing mutations. Monoallelic mutations in genes with dominant inheritance were also considered as disease-causing mutations.

### Statistical analysis

Student’s t test or Chi-square test (two-tailed) was used to compare two groups. A p value of 0.05 or less was considered significant.

## Results

Hereditary hypophosphatemia were diagnosed in 23 patients from 15 families: 17 children (mean age at diagnosis 4.9±1.9 years, age range 1.3–8.9 years), and 6 adults (mean age at diagnosis 34.2±4.5 years, age range 28–40 years). All the children had clinical and laboratory features of rickets such as genu varum deformity, metaphyseal X-ray findings, and/or fractures. The X-ray images of metaphyseal widening and irregularity, cupping and fraying of the metaphyseal region, haziness of cortical margins and epiphysis, and enlargement of the space between the epiphysis and metaphysis were present in all HR children except for X-3 and X-4. Loss of teeth and gum abscess were observed in case IX-3. Cases III-3, VI-4, and VII-3 had undergone a surgical operation for correction of severe lower limb deformities. Bone pain was not recorded in any of the cases.

All the adults were diagnosed based upon relevant clinical (bone pain, walking difficulty, muscle weakness) and laboratory findings (hypophosphatemia, mild-moderate elevated PTH, increased renal phosphate loss). Ten children (58.8%, 10/17) and five adults (83.3%, 5/6) had short stature. Although affected parents had short stature, genu varum, and/or bone fractures after minor trauma starting from childhood, none was diagnosed previously. Genetic analysis identified disease-causing gene mutations in 22 subjects (21 patients and 1 asymptomatic sibling) from 13 families: 12 different *PHEX* mutations in 20 patients from 12 families, and two different *SLC34A3* mutations in two compound heterozygous siblings, one of whom was asymptomatic. No mutation was identified in two patients from two different families.

### *PHEX* mutations

**Fig 1:**
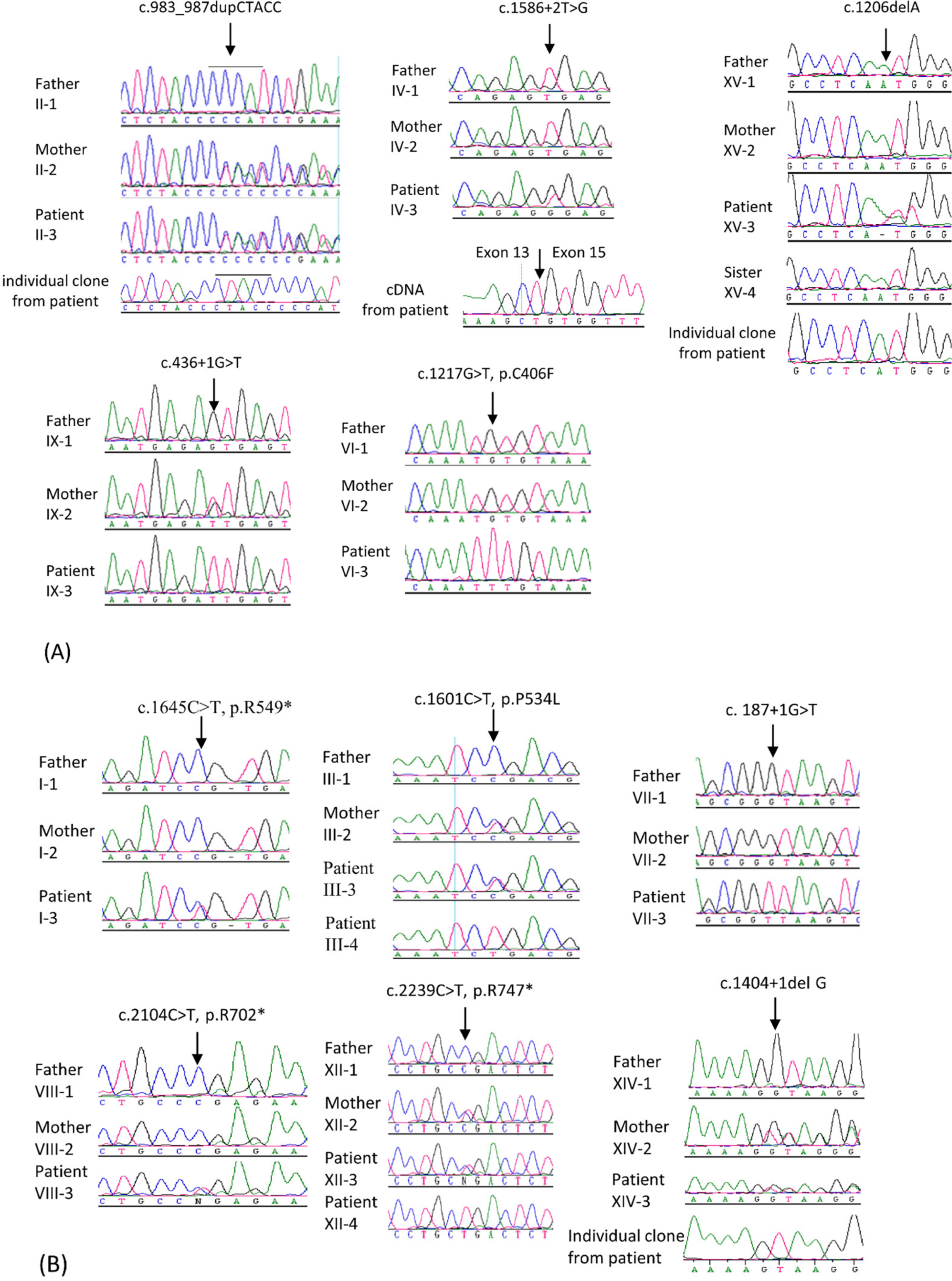
Sequence analysis of *PHEX* in patients with hereditary hypophosphatemia. (A) Five novel *PHEX* mutations. c.983_987dupCTACC (patient II-3 and her mother) and c.436+1G>T (patient IX-3 and his mother) are inherited mutations from mother; c.1586+2T>G (patinet IV-3), c.1206delA (patient XV-3), and c.1217G>T (patientVI-3) are de novo mutations not present in parents. The c.1586+2T>G results in exon 14 skipping. (B) Six previously reported *PHEX* mutations. c.1645C>T, c. 187+1G>T, and c.2104C>T are de novo mutations. c.1601C>T, c.2239C>T, and c.1404+1del G are inherited mutations transmitted from mother. Mutation is indicated by an arrow.

Six novel *PHEX* mutations were identified in 9 patients from 6 families: c.983_987dupCTACC, c.1586+2T>G, c.1206delA, c.436+1G>T, c.1217G>T (p.C406F), and a large deletion missing exon 16–22 (Fig 1A and Table 2). Although c.436+1G>T is a novel mutation, c.436+1G>A and c.436+1G>C have been reported previously in a Chinese and US patient, respectively [36, 37]. The c.1217G>T (p.C406F) is located at the same position as our previously identified mutation c.1217G>A (p.C406Y) in a Turkish patient [30]. Six previously reported *PHEX* mutations were found in 11 patients from 6 families: c.1645C>T (p.R549*) [38], c.1601C>T (p.P534L) [38], c. 187+1G>T [39], c.2104C>T (p.R702*) [38], c.2239C>T (p.R747*) [40], and c.1404+1del G (direct submission to *PHEX* mutation database: http://www.phexdb.mcgill.ca/) (Fig 1B and Table 2). The c.1586+2T>G, c.1206delA, c.1217G>T, c.1645C>T, c. 187+1G>T, and c.2104C>T were not present in the parents and therefore were de novo mutations, accounting for 50% of identified *PHEX* mutations. The effect of splice donor site mutation c.1586+2T>G on mRNA splicing was investigated by RT-PCR analysis of cDNA from patient’s peripheral lymphocytes. As shown in Fig 1A, the mutation resulted in exon 14 skipping. The c.1586+1G>A was reported previously without annotation of splicing consequence [2]. Based on our current study, the mutation is likely result in exon 14 skipping as well. Most of the mutations were splice site mutations, deletions or duplication, or nonsense mutations. These mutations are predicted to cause frameshift, resulting in either truncated protein or nonsense-mediated decay. Only two missense mutations were identified: c.1601C>T (reported) and c.1217G>T (novel). We performed *in silico* analysis of these two missense mutations as well as two previously reported mutations that were located in the same codon as c.1217G>T (c.1217G>A, p.C406Y and c.1216T>C, p.C406R) by four web-based programs. As shown in Table 3, all of them were predicted to be disease-causing mutations.

**Table 2:** Mutational analysis of patients with hereditary hypophosphatemia.

Family	Subjects	Gene	Location	Nucleotide change	Amino acid change	Zygocity	Mutation
I	I-1 Father	*PHEX*		Wild-type			
	I-2 Mother	*PHEX*		Wild-type			
	I-3 Patient	*PHEX*	Exon 15	c.1645C>T	p.R549*	Het	De novo, reported
II	II-1 Father	*PHEX*					
	II-2 Mother	*PHEX*	Exon 9	c.978_982dupCTACC	H329Tfs*3	Het	Novel
	II-3 Patient	*PHEX*	Exon 9	c.978_982dupCTACC	H329Tfs*3	Het	Novel
III	III-1 Father	*PHEX*		Wild-type			
	III-2 Mother	*PHEX*	Exon 15	c.1601C>T	p.P534L	Het	Reported
	III-3 Patient	*PHEX*	Exon 15	c.1601C>T	p.P534L	Het	Reported
	III-4 Patient	*PHEX*	Exon 15	c.1601C>T	p.P534L	Hemi-zygous	Reported
IV	IV-1 Father	*PHEX*		Wild-type			
	IV-2 Mother	*PHEX*		Wild-type			
	IV-3 Patient	*PHEX*	Splice donor site at intron 14, Exon 14 skipping	c.1586+2T>G	Frameshift	Het	De novo, novel (c.1586+1G>A reported previously)
V	V-1 Father	*PHEX*	Exon 16–22	g.22,215,887–22,395,767del(179880 bp deletion)		Hemi-zygous	Reported but not characterized previously
	V-2 Mother	*PHEX*		Wild-type			
	V-3 Patient	*PHEX*	Exon 16–22	g.22,215,887–22,395,767del(179880 bp deletion)	Truncated	Het	Reported but not characterized previously
VI	VI-1 Father	*PHEX*		Wild-type			
	VI-2 Mother	*PHEX*		Wild-type			
	VI-3 Sister	*PHEX*		Wild-type			
	VI-4 Patient	*PHEX*	Exon 11	c.1217G>T	p.C406F	Hemi-zygous	De novo, novel (c.1217G>A, p.C406Y; and c.1216T>C, p.C406R reported previously)
VII*	VII-1 Father	*PHEX*		Wild-type			
	VII-2 Mother	*PHEX*		Wild-type			
	VII-3 Patient	*PHEX*	Splice donor site at intron 2	c. 187+1G>T	Frameshift	Hemi-zygous	De novo, reported
VIII	VIII-1 Father	*PHEX*		Wild-type			
	VIII-2 Mother	*PHEX*		Wild-type			
	VIII-3 Patient	*PHEX*	Exon 21	c.2104C>T	p.R702*	Het	De novo, reported
IX	IX-1 Father	*PHEX*		Wild-type			
	IX-2 Mother	*PHEX*	Splice donor site at intron 4	c.436+1G>T	Frameshift	Het	Novel (c.436+1G>A and c.436+1G>C reported previously)
	IX-3 Patient	*PHEX*	Splice donor site at intron 4	c.436+1G>T	Frameshift	Hemi-zygous	Novel (c.436+1G>A and c.436+1G>C reported previously)
X	X-1 Father	*SLC34A3*	Splice donor site at intron 12	c.1335+2T>A	Frameshift	Het	Novel
	X-2 Mother	*SLC34A3*	Exon 13	c.1639_1652delCGCTCCTGGGCCTG	R547Afs*41	Het	Novel
	X-3 Patient	*SLC34A3*	Splice donor site at intron 12 and Exon 13	c.1335+2T>A and c.1639_1652delCGCTCCTGGGCCTG	Frameshift	Compound Het	Novel
	X-4 Sister	*SLC34A3*	Splice donor site at intron 12 and Exon 13	c.1335+2T>A and c.1639_1652delCGCTCCTGGGCCTG	Frameshift	Compound Het	Novel
XI	XI-1 Father			Wild-type			
	XI-2 Mother			Wild-type			
	XI-3 Patient			No mutation detected			
XII	XII-1 Father	*PHEX*		Wild-type			
	XII-2 Mother	*PHEX*	Exon 22	c.2239C>T	p.R747*	Het	Reported
	XII-3 Patient	*PHEX*	Exon 22	c.2239C>T	p.R747*	Hemi-zygous	Reported
	XII-4 Patient	*PHEX*	Exon 22	c.2239C>T	p.R747*	Het	Reported
XIII	XIII-1 Father			Wild-type			
	XIII-2 Mother			Wild-type			
	XIII-3 Patient			No mutation detected			
XIV	XIV-1 Father	*PHEX*		Wild-type			
	XIV-2 Mother	*PHEX*	Splice donor site at intron 12	c.1404+1delG	Frameshift	Het	Reported
	XIV-3 Patient	*PHEX*	Splice donor site at intron 12	c.1404+1delG	Frameshift	Het	Reported
XV	XV-1 Father	*PHEX*		Wild-type			
	XV-2 Mother	*PHEX*		Wild-type			
	XV-3 Patient	*PHEX*	Exon 11	c.1206del A	Q402Hfs*6	Het	De novo, novel
	XV-4 Sister	*PHEX*		Wild-type			

*from a consanguineous family

**Table 3:** *In silico* prediction of single nucleotide variations in the *PHEX* gene.

SNV NM_000444	MAF (minor allele frequency)	SIFT (score)	PROVEAN (score)	PolypPhen-2 (score)	Mutation Taster
c.1601C>T, p.P534L	neither found in ExAC nor 1000G	damaging (0.003)	deleterious (-6.89)	probably damaging (1.00)	disease causing
c.1217G>T, p.C406F	neither found in ExAC nor 1000G	damaging (0.000)	deleterious (-10.87)	probably damaging (1.00)	disease causing
c.1217G>A, p.C406Y	neither found in ExAC nor 1000G	damaging (0.000)	deleterious (-10.87)	probably damaging (1.00)	disease causing
c.1216T>C, p.C406R	neither found in ExAC nor 1000G	damaging (0.000)	deleterious (-11.86)	probably damaging (1.00)	disease causing

**Fig 2:**
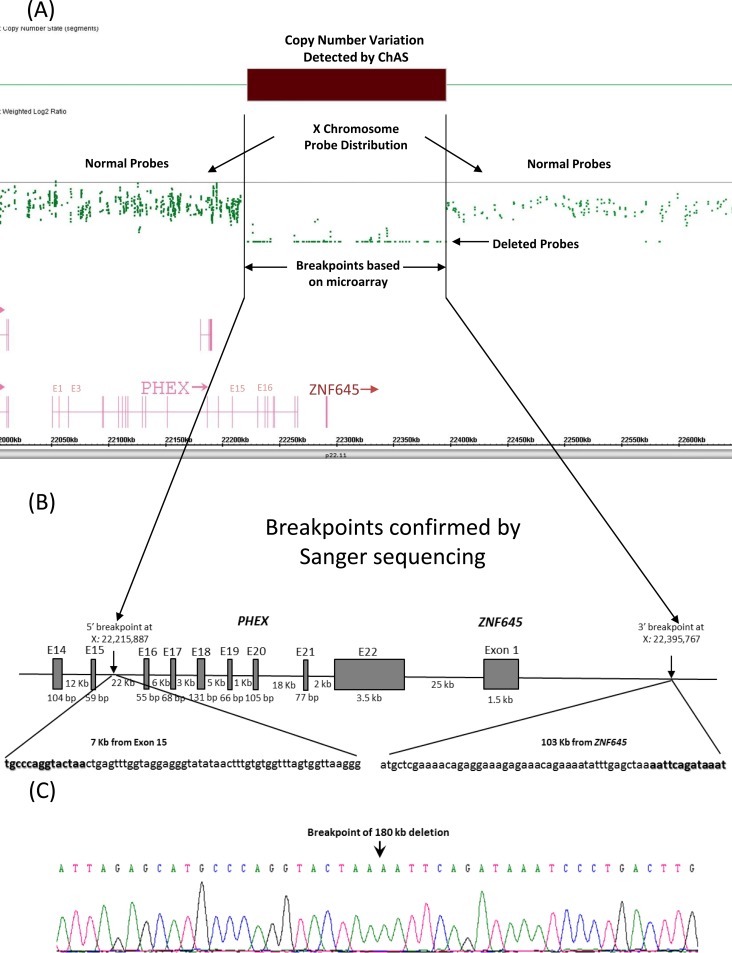
Characterization of a large deletion of *PHEX* in patient V-3. (A) Genechip cytogenetics array results. The top is showing weighted log2 ratios plot showing a normal copy number state for the X chromosome. The deletion is indicated by a dark bar and enclosed in the black lines. More than 70 SNP probes are present in the deleted region and indicated by an arrow. (B) Schematic representation of 179,880 bp deletion of *PHEX* and *ZNF645*. The 5’ breakpoint is located in the intron 15 of *PHEX* and 7 kb from exon 15. The 3’ breakpoint point is 103 kb from *ZNF645*, resulting in the deletion of exon 16–22 of PHEX and entire *ZNF645*. The 5’ and 3’ undeleted nucleotide sequences are highlightted in bold. Deletion analysis is based on GRCh37/hg19. (C) Electropherogram of the breaking point. The DNA fragment was amplified by PCR using primers flanking the 5’ and 3’ deletion point. The breakpoint is indicated by an arrow.

**Fig 3:**
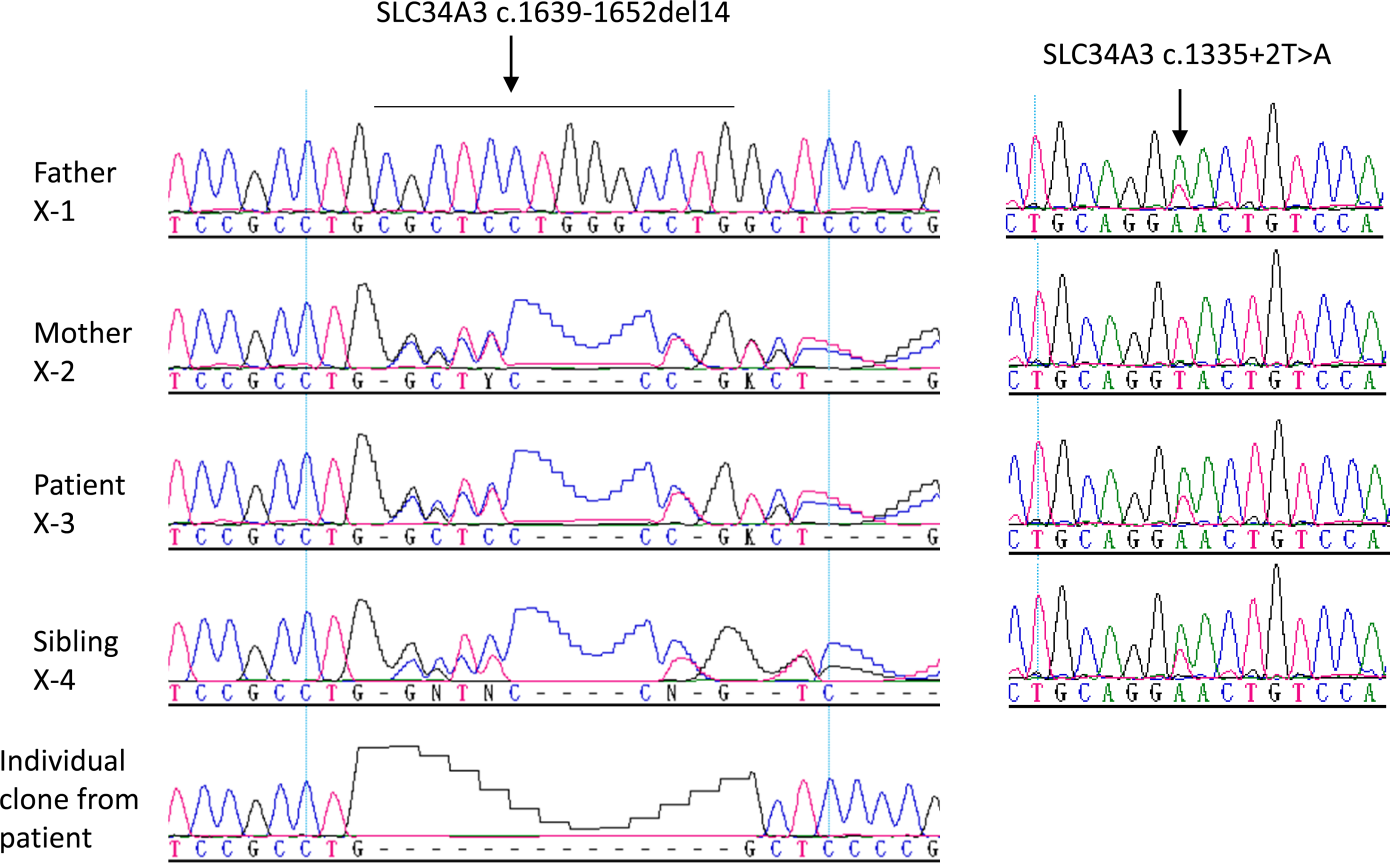
Sequence analysis of *SLC34A3* in a patient with hereditary hypophosphatemia. A novel heterozygous mutation c.1639-1652del14 is present in the mother, patient, and his sister. A novel heterozygous splice donor site mutation c.1335+2T>A is present in the father, patient, and his sister. Compound heterozygous *SLC34A3* mutations are carried by both patient and his sister. Mutation is indicated by an arrow.

We could not amplify by PCR the region containing *PHEX* exon 16–22 from affected father of index patient (V-3) from family V whereas the same region was amplified from the female patient and her mother’s DNA, suggesting the possible presence of a large deletion. We next performed a copy number analysis and identified the deleted region (Fig 2A). Based on the 5’ and 3’ undeleted probe sequences closest to the deleted region, we performed chromosome walking to identify the breakpoint. As shown in Fig 3B and 3C, the 5’ breakpoint was located in the intron 15 and 7 kb from the exon 15 of the *PHEX* gene (X: 22,215,887); the 3’ breakpoint was 103 kb from the *ZNF645* gene and 130 kb from the *PHEX* gene (X: 22,395,767), resulting in the deletion of 179,880 bp DNA fragment. Single TAA is present at the 5’ breakpoint and immediately before the 3’ breakpoint, which may be used for homologous recombination (Fig 2B and 2C).

The mean age at diagnosis of 14 children with *PHEX* mutations (6 males, 8 females) were 3.3±1.1 (range, 1.3–5.0) years. The mean SD score of height was -2.26±1.46 (-3.89–1.18) and 71.4% of them (10/14) had short stature. All had clinical findings compatible with rickets. Laboratory tests of all subjects revealed typical features of XLHR: normal levels of calcium (9.5±0.5 mg/dL; range 8.5–10.3 mg/dL), low levels of serum phosphate (2.7±0.5 mg/dL; range, 2.0–3.6 mg/dL), TRP (69.8±8.0%, range, 59.9–79.6%), and TmP/GFR (1.88±0.36; range, 1.31–2.48), increased levels of ALP (598±175 IU/L; range, 364–925), and normal/mildly increased levels of PTH (84.5±34.8 pg/mL; range, 31.9–154.7).

The mean age at diagnosis of adults with *PHEX* mutations (1 male, 5 females) was 34.2±4.5 years (range, 28–40 years). Mean SD score of height was -2.92±0.93 (range, -3.95– -1.46) and 5 of them (83.3%) were short. All of these patients had normal calcium (10.1±0.4 mg/dL; range, 8.9–10.1 mg/dL, normal: 8.5–10.5 mg/dL), hypophosphatemia (2.0±0.2 mg/dL; range, 1.7–2.3 mg/dL, normal: 2.7–4.5 mg/dL), normal urine calcium/creatinine ratio (range, 0.04–0.15 mg/mg; normal: <0.2), and decreased TmP/GFR (1.76±0.31; range, 1.46–2.2, normal: 2.5–3.6). ALP values were in normal range in all except one (111 ± 22 IU/L, range, 81–143; normal: 41–120). Moreover, varying levels were found for PTH (79.8±34.4 pg/mL; range, 40–130 pg/mL; normal: 11.1–79.5 pg/mL) and TRP (87.4±14.4%; range 69.9–97%; normal: >85%) (Table 1).

When the patients with *PHEX* mutation were evaluated according to the type of inheritance (de novo or inherited), height SD scores and the frequency of short stature were significantly lower in the inherited group (Table 4). Although patients with an inherited mutation was younger at the time of diagnosis when compared to the patients with a de novo mutation, this difference was not statistically significant. The patients were successfully treated with calcitriol and phosphate.

**Table 4:** Clinical and laboratory characteristics of children with de novo or inherited *PHEX* mutations.

Parameters	De novo (n = 6)	Inherited (n = 8)	p Value
Age (years)	3.8 (3.1 / 4.6)	2.9 (2.2 / 4.1)	0.228
Height SDS	-3.60 (-3.79 /-3.30)	-1.51 (-2.10 / -0.79)	0.001*
Short stature	6	4	0.035*
Ca (mg/dL)	9.6 (9.4 / 9.9)	9.5 (8,5 / 9.9)	0.755
P (mg/dL)	2.6 (2.3 / 3.1)	2.8 (2.2 / 3.0)	0.983
ALP (IU/L)	629 (526 / 853)	532 (406 / 656)	0.228
25-(OH) vitamin D (ng/mL)	31.6 (17.1 / 56.0)	33.7 (24.5 / 67.0)	0.755
PTH (ng/mL)	86.5 (69.2 / 142.1)	66.7 (49.2 / 106)	0.181
TRP	73.7 (58.2 / 78.8)	71.6 (63.7 / 74.9)	0.662
TmP/GFR	1.85 (1.54 / 2.05)	1.99 (1.53 / 2.42)	0.662

*Statistically significant

### *SLC34A3* mutations

Two novel *SLC34A3* compound heterozygous mutations were found in the index case from Family X (X-3) and his asymptomatic sister (X-4): a splice donor site mutation c.1335+2T>A and a 14 bp deletion c.1639_1652delCGCTCCTGGGCCTG (Fig 3 and Table 2). A heterozygous c.1335+2T>A and c.1639_1652delCGCTCCTGGGCCTG were carried by his non-consanguineous father and mother, respectively. The index case, a 5-year-old boy was first evaluated by a nephrologist (SK) due to nephrocalcinosis. Hypophosphatemia and elevated ALP were also observed in addition to hypercalciuria (0.23 mg/mg urine calcium/creatinine ratio, normal <0.20) but were not considered clinically significant initially due to lack of other rachitic findings (Table 1). He had no additional symptoms or findings consistent with rickets. Oral phosphate treatment was started at the age of 14 years following the genetic diagnosis. Consequently, ALP level decreased from 483 U/L to 258 U/L (normal, 74–390). His 6.9-year-old asymptomatic sister had the same mutation. Detailed investigation revealed normal clinical and laboratory variables except for the intermittent mild hypophosphatemia (3.6–4.4 mg/dL, normal 3.7–5.6) and increased urine calcium/creatinine ratio (0.7 mg/mg). She did not require phosphate treatment during 2 years of follow-up. Their father (Case X-1) had recurrent nephrolithiasis but mother (Case X-2) was free of symptoms. Biochemical profiles were normal in both parents.

### Families without known mutations

No mutation was detected in the patients from families XI and XIII. Copy number analysis of whole genome did not reveal any large deletions as well.

Case XI-3, a 2.5-year-old boy, was admitted due to skeletal deformities of his hands and feet. His parents were not relatives. Physical examination revealed a normal height (SD score -0.4), prominent widening of wrists, and genu varum. Hypophosphatemia, increased ALP level and urine phosphate excretion, normal calcium and PTH levels, and rachitic X-ray findings were found (Table 1). Vitamin D supplement due to low 25-hydroxyvitamin D3 level did not result in improvement. Calcitriol and neutral phosphate treatment with the diagnosis of HR resulted in resolution of rickets and normalization of laboratory variables.

The other patient (XIII-3) was a 7.5-year-old boy who was evaluated due to inability to walk during the past 1 year. Physical examination was normal other than widening of wrists and genu varum deformities. Laboratory and skeletal X-ray findings were consistent with HR (Table 1). Calcitriol and neutral phosphate were started, and clinical and laboratory improvement were achieved within the first year. His treatment was gradually reduced and stopped when he was 13 years old. The most recent follow-up without treatment was made when he was 21 years old. Physical examination revealed a height of 175.5 cm (-0.19 SDS) and no skeletal abnormalities. Laboratory studies showed normal calcium (9.3 mg/dL, normal 8.8–10.5), ALP (192 IU/L, normal 93–309), and 25- hydroxyvitamin D3 (20.6 ng/mL, normal >20), but low serum phosphate (1.96 mg/dL, normal 2.7–4.5), mildly increased PTH (96 pg/mL, normal 11.1–79.9), normal TRP (91.0%, normal <85%), decreased TmP/GFR (1.78, normal 2.5–4.5), and a normal urine calcium/creatinine ratio (0.01 mg/mg creatinine, normal <0.20).

## Discussion

In the present study, we investigated 23 patients from 15 unrelated families with hereditary hypophosphatemia. Disease-causing mutations were found in 21 patients and one asymptomatic sibling (carrying compound heterozygous *SLC34A3* mutations) including 8 novel mutations: 6 in *PHEX*, and 2 in *SLC34A3*. De novo *PHEX* mutations were found in 6 out of 12 families. Together with our previous studies [30–32], we have investigated 50 patients from 31 unrelated families with hereditary hypophosphatemia, representing the largest series of patients from Turkish population. Among the 31 families, *PHEX*, *FGF23*, *CLCN5*, and *SLC34A3* mutations was found in 26 (83.9%), 1 (3.2%), 1 (3.2%), and 1 (3.2%) families, respectively. The overall mutation detection rate is about 94% (29/31) and *PHEX* mutation is the most common genetic defect in the Turkish patients. Other mutations are rare such as *FGF23*, *DMP1*, *ENPP1*, *SLC34A1*, *SLC34A3*, and *CLCN5*. In fact, we have not detected *DMP1*, *ENPP1*, or *SLC34A1* mutation in our patients. Our data are similar to those from other parts of the would such as Italy [2], Denmark [5] and US [6].

The *PHEX* gene is located on the X chromosome and consists of 22 short exons encoding a protein of 749 amino acids [41]. It is primarily expressed in osteoblasts and osteocytes, and plays an important role in phosphate metabolism [41]. An inactivating mutation of *PHEX* leads to increased serum FGF23 level and thus renal phosphate loss. Most *PHEX* mutations found in the current study are either splice site or insertion/deletion mutations, resulting in either truncated proteins devoid of normal function or truncated mRNA likely going through nonsense-mediated decay [42]. The delay in diagnosis of our patients with de novo *PHEX* mutations is likely due to lack of family history and awareness by their physicians, which results in treatment delay and more severe growth retardation. We could not detect mutation in two patients from 2 families, which may indicate possible existence of a promoter mutation in the screened genes or a novel disease-causing gene, which needs to be investigated further.

We have characterized another *PHEX* deletion with identification of the breakpoint (g.22,215,887–22,395,767del). The large *PHEX* deletion missing exon 16–22 has been reported previously but the breakpoint has never been investigated [5, 43]. It is not clear whether previously reported cases have the same breakpoint or not. Interestingly, TAA repeats are also present in the 5’ breakpoint of our previously characterized 40 kb *PHEX* deletion [31]. Unlike the 40 kb *PHEX* deletion which has 13 TAA tandem repeats present before the 3’ breakpoint, only one TAA repeat is present before the 3’ breakpoint in the current deletion, suggest that the deletion mediated by homologous recombination may not need multiple tandem repeats. The entire *ZNF645* is also deleted together with exon 16–22 of the *PHEX* gene. *ZNF645* is a RING finger protein and function as an E3 ubiquitin-protein ligase. It may play a role in human sperm production and quality control [44].

The c.1335+2T>A and c.1639_1652del14 mutations in *SLC34A3* are predicted to cause frameshift and result in loss-of-function of the gene. Given the autosomal recessive inheritance of HHRH, both patient and his sister should have the disease. The apparent phenotype-genotype discordance in the patient’s sister is not clearly known. Various genetic and/or epigenetic factors may contribute to the phenotype variation. A recent meta-analysis have demonstrated that nearly 25% of individuals with homozygous or compound heterozygous *SLC34A3* mutations have no rickets/osteomalacia, and 50% of individuals without renal calcifications (nephrolithiasis or nephrocalcinosis), reflecting the heterogeneity in clinical presentations caused by *SLC34A3* mutations [45].

In summary, we have systemically investigated genetic defects in Turkish patients with hereditary hypophosphatemia. *PHEX* mutation is the most common genetic defect accounting for 84% of the disease. De novo *PHEX* mutations are common and often contribute to the delay in diagnosis and treatment. The early diagnosis of new-born family members may be possible by screening asymptomatic children who have family history of disease-causing mutations or the knowledge of frequent de novo *PHEX* mutations in the population. This will enable early treatment and a better growth outcome.
